# Evidence for pleural epithelial-mesenchymal transition in murine compensatory lung growth

**DOI:** 10.1371/journal.pone.0177921

**Published:** 2017-05-19

**Authors:** Alexandra B. Ysasi, Willi L. Wagner, Cristian D. Valenzuela, Arne Kienzle, Andrew B. Servais, Robert D. Bennett, Akira Tsuda, Maximilian Ackermann, Steven J. Mentzer

**Affiliations:** 1 Laboratory of Adaptive and Regenerative Biology, Brigham & Women’s Hospital, Harvard Medical School, Boston, Massachusetts, United States of America; 2 Institute of Functional and Clinical Anatomy, University Medical Center of the Johannes Gutenberg-University, Mainz, Germany; 3 Molecular and Integrative Physiological Sciences, Harvard School of Public Health, Boston, Massachusetts, United States of America; University of South Alabama Mitchell Cancer Institute, UNITED STATES

## Abstract

In many mammals, including rodents and humans, removal of one lung results in the compensatory growth of the remaining lung; however, the mechanism of compensatory lung growth is unknown. Here, we investigated the changes in morphology and phenotype of pleural cells after pneumonectomy. Between days 1 and 3 after pneumonectomy, cells expressing α-smooth muscle actin (SMA), a cytoplasmic marker of myofibroblasts, were significantly increased in the pleura compared to surgical controls (p < .01). Scanning electron microscopy of the pleural surface 3 days post-pneumonectomy demonstrated regions of the pleura with morphologic features consistent with epithelial-mesenchymal transition (EMT); namely, cells with disrupted intercellular junctions and an acquired mesenchymal (rounded and fusiform) morphotype. To detect the migration of the transitional pleural cells into the lung, a biotin tracer was used to label the pleural mesothelial cells at the time of surgery. By post-operative day 3, image cytometry of post-pneumonectomy subpleural alveoli demonstrated a 40-fold increase in biotin^+^ cells relative to pneumonectomy-plus-plombage controls (p < .01). Suggesting a similar origin in space and time, the distribution of cells expressing biotin, SMA, or vimentin demonstrated a strong spatial autocorrelation in the subpleural lung (p < .001). We conclude that post-pneumonectomy compensatory lung growth involves EMT with the migration of transitional mesothelial cells into subpleural alveoli.

## Introduction

Lung regeneration occurs in a variety of adult mammals [1], including humans [2], after the surgical removal of one lung (pneumonectomy). The remaining lung becomes larger; however, the expansion of the lung is not simple isotropic expansion as there is a commensurate increase in lung weight [3] and cell number [4]. Moreover, advanced morphometric techniques have demonstrated an increase in the number of alveoli [5]. These observations indicate that post-pneumonectomy lung growth involves extensive remodeling of the lung microarchitecture [6,7].

Recent attempts to define a mechanism for neoalveolarization have identified several morphologic clues. First, post-pneumonectomy lung growth is not associated with aggregates of proliferative cells; rather, lung growth is associated with an increase in septal thickness [6,7]. Second, early morphologic changes include a retraction of alveolar septa and an increase in alveolar duct diameter. Ysasi et al have suggested that alveolar growth involves a repartitioning of the dilated alveolar ducts [7].

A cell type potentially involved in the re-partitioning of the alveolar duct is the myofibroblast. Characterized by the cytoplasmic expression of α-smooth muscle actin (SMA) and the production of extracellular matrix components, myofibroblasts have been spatially associated with the alveolar stage of lung development [8,9]; specifically, histologic evidence has implicated the myofibroblast in the “lifting” of alveolar septa and the partitioning of alveolar ducts [10]. In the remaining lung after pneumonectomy, there is an increase in pleural SMA expression within 24 hours. Single-cell analysis has demonstrated the apparent migration of the deformation-induced SMA^+^ cells into regenerating alveolar ducts [11].The increased numbers of pleural-derived SMA^+^ cells after pneumonectomy suggests the induction of epithelial-mesenchymal transition (EMT); however, direct evidence for post-pneumonectomy pleural EMT has been lacking.

Here, we used whole-lobe image cytometry and scanning electron microscopy (SEM) to examine the visceral pleura post-pneumonectomy. We provide direct evidence that compensatory lung growth involves pleural EMT with the migration of transitional mesothelial cells into subpleural alveoli.

## Methods

### Animals

The care of the animals was consistent with guidelines of the American Association for Accreditation of Laboratory Animal Care (Bethesda, MD, USA) and approved by our Institutional Animal Care and Use Committee (Brigham & Women's Hospital). Male mice, eight to ten-week old wild type C57BL/6 (Jackson Laboratory, Bar Harbor, ME, USA) were anesthetized as previously described [12]. Briefly, mice are kept in social housing (up to 4 littermates per cage) in individually ventilated isolation cages, with continuous access to water and standard laboratory rodent diet pellets. Post-operatively, the mice are kept individually to prevent fighting. Rodent nestlets and cardboard homes are provided as environmental enrichment.

### Pneumonectomy

Each animal undergoing pneumonectomy was ventilated on a Flexivent (SciReq, Montreal, QC Canada) at ventilator settings of 200 breaths/min, 10 ml/kg, and PEEP of 2 cmH_2_O with a pressure limited constant flow profile [12]. A left fifth intercostal space thoracotomy provided exposure for hilar ligation and left pneumonectomy. Postoperatively, the animal was weaned from mechanical ventilation and maintained on supplemental oxygen until normal spontaneous ventilation was observed. Sham thoracotomies—using the same anesthetic technique, an identical incision, and closure without surgical manipulation of the left lung—were included in each experimental condition. In some mice, plombage or phrenic nerve transection was performed as previously described [13]. Postoperatively, the animal was weaned from mechanical ventilation and maintained on supplemental oxygen until normal spontaneous ventilation was observed.

### Plombage

Prior to all measurements, the pressure transducers and ventilator tubing of the FlexiVent (Scireq, Montreal, QC) were calibrated by the two collected-point method. The volume and resistance of the endotracheal tube were then calibrated with open and closed measurments. After intubation, the mice were transferred to the FlexiVent system (SciReq) for pulmonary mechanics studies. The animals were ventilated at a rate of 300/minute and allowed to acclimate to the ventilator for two minutes before standardization of the volume history with 3 consecutive recruitment maneuvers (“TLC” by SciReq; 3 sec ramp to 30cm H_2_O)) followed by standard volume measurements. Standard thoracotomy and pneumonectomy was performed (above). Post-pneumonectomy, the volume history was again standardized with 3 consecutive “TLC” recruitment maneuvers. Pulmonary volumes were compared pre- and post-pneumonectomy for calculation of plombage volume (Bone wax, Ethicon, Somerville, NJ). The plombage wax was inserted with standard closure of the thoracotomy (above). Using this technique, microCT images showed no mediastinal shift or functional compression of the contralateral lung. In a subset of mice, normal mechanics was confirmed with pulmonary mechanics studies [14,15]. Subsequent to baseline measurements ventilation was briefly stopped and the animal passively exhaled to functional residual capacity; an 8 second multifrequency (0.5–19.5 Hz) oscillatory signal (Prime-8 by SciReq) was delivered with ventilation resumed at the completion of the maneuver.

### Phrenic nerve transection

In phrenic nerve transection mice, the standard thoracotomy and pneumonectomy was performed (above). At the conclusion of the pneumonectomy, the phrenic nerve was identified on the left lateral pericardium. The nerve was mobilized with gentle traction. Diaphragmatic tenting confirmed the nerve identity. The phrenic nerve was transected sharply at the level of the pulmonary vein. A recruitment maneuver (named “TLC” by SciReq) was performed while closing the thoracotomy. The animals were removed from the ventilator and extubated upon recovery of spontaneous ventilation. Fiducials were placed in the mid-clavicular line at the costal margins. Digital video of the fiducial movement was routinely recorded on emergence from anesthesia. Asymmetric ventilation was confirmed by video analysis as previously described [13]. The animals were recovered with supplemental oxygen and external warming; Subcutaneous Buprenorphine 2.4 ug (Hospira Inc., Lake Forest, IL) was administered twice daily for 48 hours.

### Biotinylation

At the conclusion of the pneumonectomy, the left hemithorax was instilled with 200ul of PBS containing EZ-link sulfo-NHS-LC-Biotin (Thermo Scientific, Waltham, MA, USA) at pH 7.8 prior to routine thoracotomy closure. The biotin was detected by avidin labeled with near-infrared (IR) Quantum Dots (Qdot800, streptavidin; Life Technologies).

### Lung fixation

After the induction of anesthesia with intraperitoneal injection of ketamine 100 mg/kg (Fort Dodge Animal Health, Fort Dodge, IA, USA) and xylazine 10 mg/kg (Phoenix Scientific, St. Joseph, MO, USA), the animal was endotracheally intubated with an 18G angiocatheter (Becton Dickinson, Franklin Lakes, NJ, USA) and ventilated with the Flexivent rodent ventilator (SciReq, Montreal, Quebec, CA). Three “TLC perturbations” (SciReq), involving a 3 sec ramp to 30 cm H20 plateau pressures, were performed. Total lung capacity (TLC) volumes were recorded. After these measurements, a midline abdominal incision was made from pubis to xyphoid process, exposing the abdominal viscera. The animal was euthanized by exsanguination through the inferior vena cava. The pleural pressures were equilibrated through a 1mm subxyphoid puncture. A median sternotomy facilitated exposure of the bilateral lungs and heart. In sequence, the left atrium, right ventricle and inferior vena cava were incised. A 22G olive-tipped cannula was inserted through the right ventricle into the pulmonary artery and the lungs were flushed with 20cc of phosphate-buffered saline at 23°C. A cervical tracheotomy was performed and the orotracheal tube replaced with a second 18G angiocatheter positioned in the distal trachea and secured with a 3–0 silk tie. The lungs were inflated to 70% TLC (based on the average of the volumes previously recorded on the Flexivent). During 70% TLC static inflation, the pulmonary artery was flushed first with 20cc of phosphate-buffered saline at 23°C then 20cc of 4% paraformaldehyde in PBS at 23°C. The lungs were maintained at 70% TLC for 1 hour while submerged in 4% paraformaldehyde in PBS at 23°C. Following the flush, the specimen was then transferred to 4% paraformaldehyde in PBS at 4°C for 24 hours. After 24 hours of fixation, 0.5-1cc of 50% OCT in PBS was injected via the trachea. Individual lobes were submerged in OCT and frozen in a mixture of acetone and dry ice. The OCT blocks were kept at -80°C for 24 hours prior to cryosectioning.

### Immunohistochemistry

Cryostat sections were obtained from lung specimens, perfused with O.C.T. compound, and snap frozen. After warming the slide to 27°C, the sections were fixed and permeabilized in acetone at 4°C. The slides were washed with buffer (PBS, 5% goat serum, 0.1% azide, 1mM MgCl_2_, 1mM CaCl_2_) and blocked with 20% goat serum, and 0.1% azide in PBS. The slides were treated with primary and detection antibodies as well as second antibody controls (no primary antibody or nonspecific polyclonal IgG). The slides were incubated for one hour at 27°C, washed 3 times and mounted with Hoechst 33342 (Sigma-Aldrich, St. Louis, MO, USA) or DAPI-containing medium (Vector Laboratories. Burlingame, CA, USA).

### Antibodies

All antibodies were obtained from commercial sources. Polyclonal antibodies included anti-αSMA (rabbit anti-mouse, Abcam, ab5694), anti-mesothelin (rabbit anti-mouse, Abbiotec, 250519), anti-vimentin (chicken anti-mouse, Abcam, ab24525), anti-claudin 5 (rabbit anti-mouse, Abcam, ab15106), anti-occludin (rabbit anti-mouse, Abcam, ab168986), anti-WT1 (rabbit anti-mouse, Abcam, ab15249). Secondary antibodies included Texas Red goat anti-rabbit IgG (H+L)(Life Technologies) and FITC goat anti-chicken IgY (H+L)(Life Technologies). Monoclonal antibodies included anti-E-cadherin (rat clone ECCD-2, IgG1, Thermo, 13–1900).

### Fluorescence microscopy

The tissue sections were imaged with a Nikon Eclipse TE2000 inverted epifluorescence microscope using Nikon objectives of 10x and 20x linear magnification with infinity correction. An X-Cite™ (Exfo, Vanier, QC, Canada) 120 W metal halide light source and a liquid light guide were used to illuminate the tissue samples. The excitation and emission filters (Chroma Technology, Bellows Falls, VT, USA) were controlled by a MAC5000 controller (Ludl, Hawthorne, NY, USA) and MetaMorph® software 7.8 (Molecular Devices, Downington, PA, USA). The fluorescence microscopy 16-bit fluorescent images were digitally recorded on a C9100-02 camera (Hamamatsu, Japan), digitally recombined and pseudocolored based on recording wavelength.

### Scanning electron microscopy (SEM)

After coating with 20–25 A gold in an argon atmosphere, the mesothelial layer was imaged using a Philips XL30 ESEM scanning electron microscope (Philips, Eindhoven, Netherlands) at 15Kev and 21μA. Stereo pair images were obtained using a tilt angle difference of 6° on a eucentric sample holder using standardized computerization.

### Transmission electron microscopy (TEM)

After cannulation of the trachea, the tissue was fixed by instillation of 2.5% buffered glutaraldehyde into the airways followed by the instillation of 50% OCT medium (Tissue Tek, Fisher Scientific) in saline. After fixation, samples of the cardiac lobe were embedded in Epon (Serva, Heidelberg, Germany). Ultrathin (700 -A°) sections were analyzed using a Leo 906 digital transmission electron microscope (Leo, Oberkochen, Germany).

### Image segmentation and cytometry

Automated 16-bit fluorescent image acquisition was performed using MetaMorph 7.8 (Molecular Devices) and the MAC5000 controller (Ludl). The multiparameter images of the section of the cardiac lobe were combined into an image stack (*.stk file). The variable-sized image stack contained positional metadata that allowed subsequent reconstruction into an image montage. The image stack was processed using standard MetaMorph filters and segmented into cell nuclei and cytoplasmic features using custom routines created using CellProfiler (Broad Institute, Cambridge, MA, USA). The segmented images from CellProfiler were reconstructed into image montages of the cardiac lobe using FCS Express 5 software (De Novo Software, Los Angeles, CA, USA). Using FCS Express 5 (De Novo), the high expressing SMA cells on the dot plot were gated and the corresponding cells in the image montage were highlighted.

### Data analysis and graphical display

The image cytometry data was converted to the FCS file standard [16] using available conversion software (GenePattern, Broad Institute). The FCS file was subsequently analyzed using hierarchical and combination gates using the FCS Express 5 (De Novo) and Cytobank (www.cytobank.org) software. Spatial autocorrelation measures were based on the image cytometry patterns obtained in the whole-lobe analysis. After separately imaging the biotin^+^, SMA^+^ and vimentin^+^ cells, the spatial autocorrelation measures were applied to the planar lobe image grid using Moran’s I as previously described [3]. Absolute migration barriers, such as alveolar airspace, were not considered. In deformation and image cytometry studies, statistical analyses were based on measurements in at least three different mice. The unpaired Student’s t-test for samples of unequal variances was used to calculate statistical significance. The data was expressed as mean ± one standard deviation. The significance level for the sample distribution was defined as p< 0.05.

## Results

### Visceral pleural mesothelium

**Fig 1:**
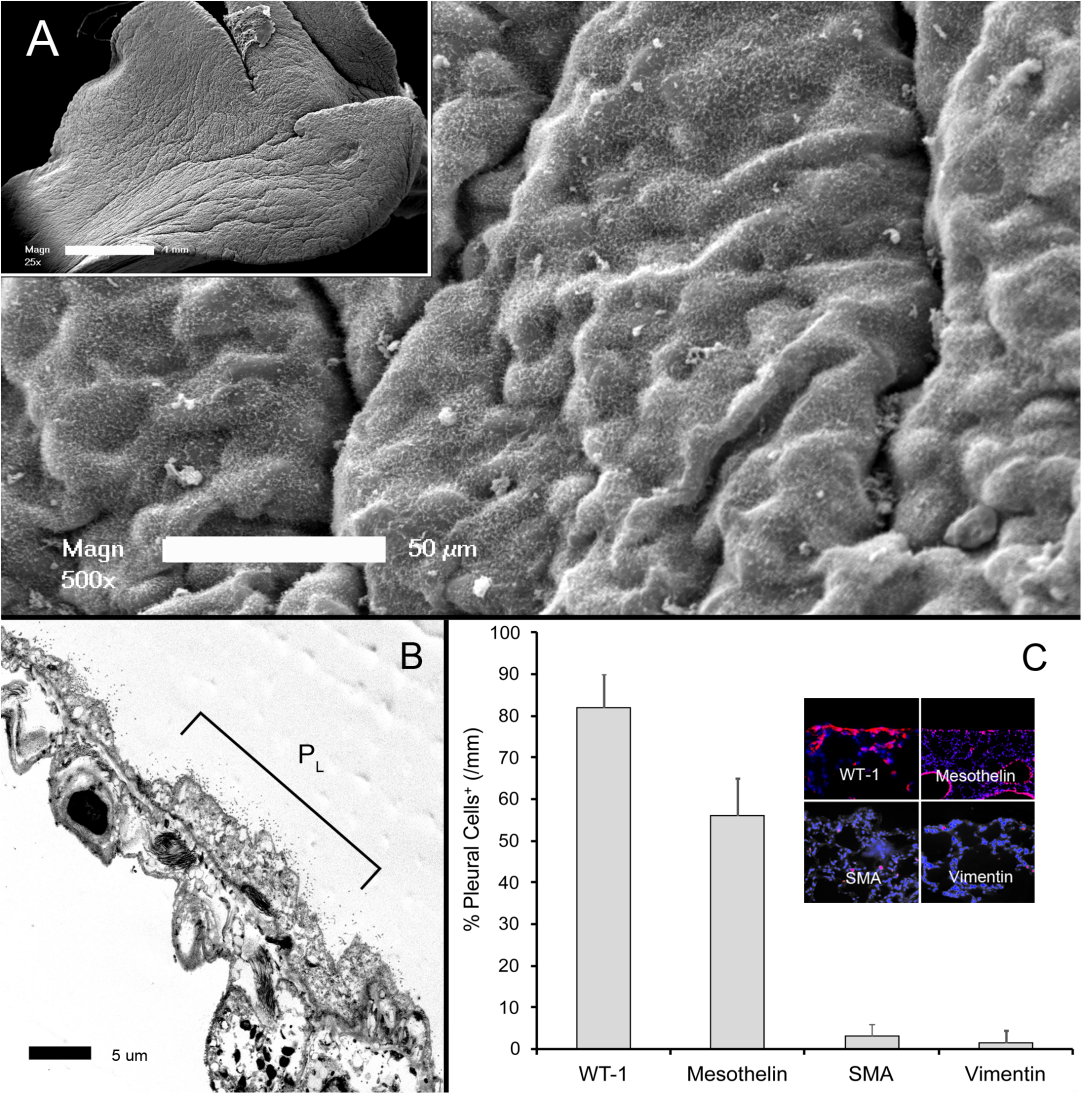
Morphology of the visceral pleural mesothelium. A) Scanning electron microscopy (SEM) of the cardiac lobe 3 days after sham thoracotomy. The pleural surface demonstrated the characteristic "bumpy" appearance of the mesothelium covered by microvilli (inset, whole lobe). B) The thin mesothelial monolayer and microvilli were apparent on transmission electron microscopy (TEM)(P_L_ = free pleural surface). C) The control mesothelium expressed WT-1 and mesothelin; only rare cells expressed the cytoskeletal proteins SMA and vimentin (inset, immunofluorescence staining). Immunostaining reflects the percentage of positive pleural cells per mm of cardiac lobe pleura.

Scanning electron microscopy (SEM) of the visceral pleura demonstrated the characteristic "bumpy" appearance in both nonsurgical and sham thoracotomy controls (Fig 1A). Microvilli were visible on both SEM and transmission electron microscopy (TEM)(Fig 1B). The intervillar glycocalyx was not visible on standard electron microscopy [17]. The normal mesothelium expressed the cell surface markers WT-1 and mesothelin, but rarely expressed the cytoskeletal proteins SMA and vimentin (Fig 1C). Both electron microscopy and immunohistochemistry demonstrated intact tight junctions.

### Deformation-induced changes in phenotype

**Fig 2:**
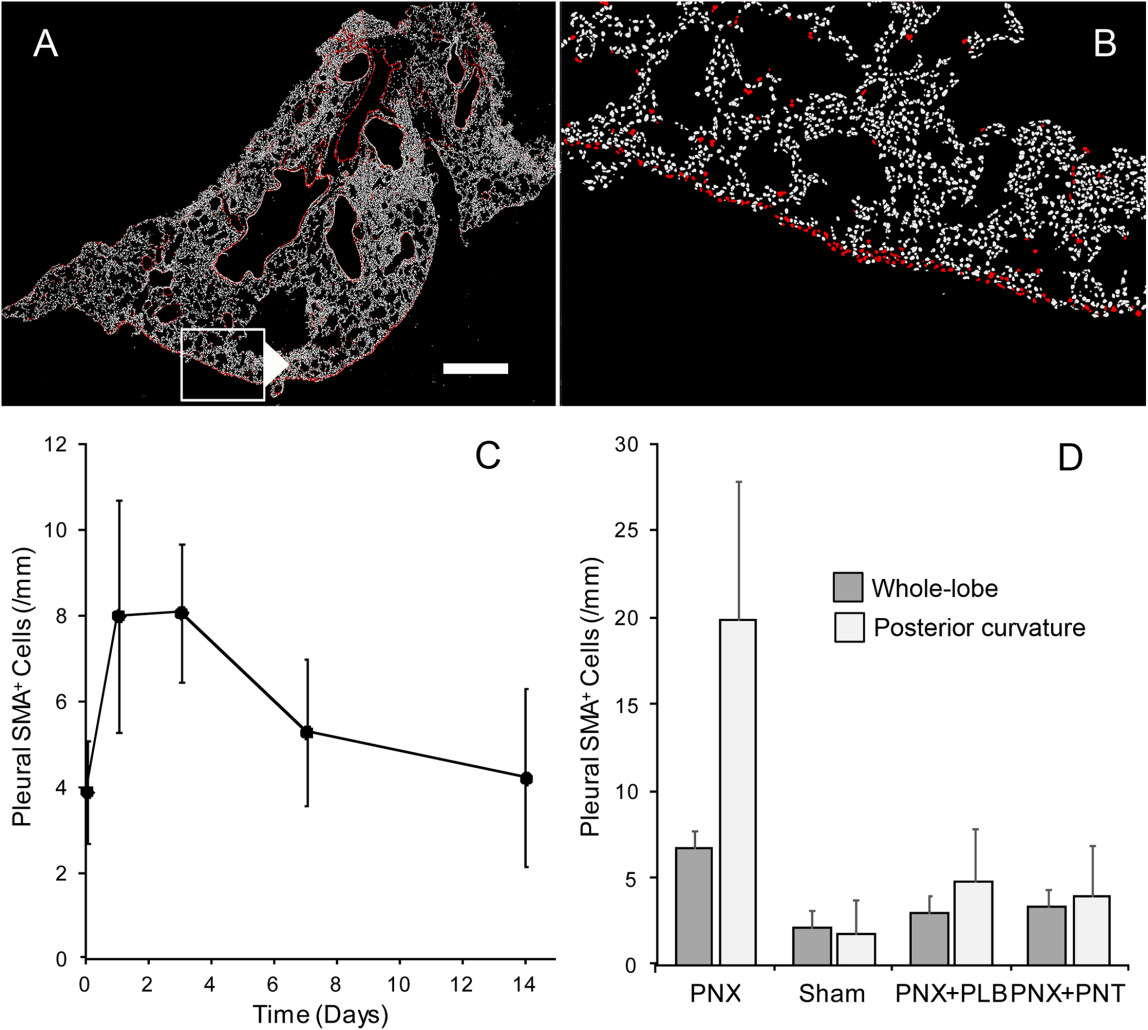
Whole-lobe multiwavelength cell scoring of the remaining cardiac lobe after left pneumonectomy. A, B) The positively curved posterior pleural surface stained for α-smooth muscle actin (SMA)(SMA = red, bar = 200um). C) Extending previous observations of SMA expression in the first 3 days after pneumonectomy [11], the most reproducible expression was on a postoperative day 3 (POD 3). D) SMA expression after pneumonectomy (PNX) was compared to conditions controlling for pleural deformation: sham thoracotomy (Sham), pneumonectomy and plombage (PNX+PLB), pneumonectomy and phrenic nerve transection (PNX+PNT)(N = 3 each condition). SMA expression after pneumonectomy mice significantly higher than in the three control conditions (p < .01). The difference was greater when the analysis was restricted to the posterior curvature of the cardiac lobe (p < .01). There was no difference between the control conditions (p>.05).

Confirming previous observations [11], whole-lobe image cytometry demonstrated increased expression of pleural SMA after pneumonectomy (Fig 2A and 2B). The percentage of SMA expression peaked between days 1 and 3, falling to near-baseline levels by 2 weeks after pneumonectomy (Fig 2C). To confirm the influence of pleural deformation on SMA induction, SMA expression was compared in three control conditions: sham thoracotomy, pneumonectomy with plombage, and pneumonectomy with phrenic nerve transection. SMA expression was significantly higher in the pneumonectomy group (p < .01)(Fig 2D).The SMA difference between pneumonectomy and control pleura was greater if the analysis was restricted to the posterior cardiac lobe (p < .001); that is, a portion of the cardiac lobe with a prominent positive curvature.

### Pleural epithelial-mesenchymal transition (EMT)

**Fig 3:**
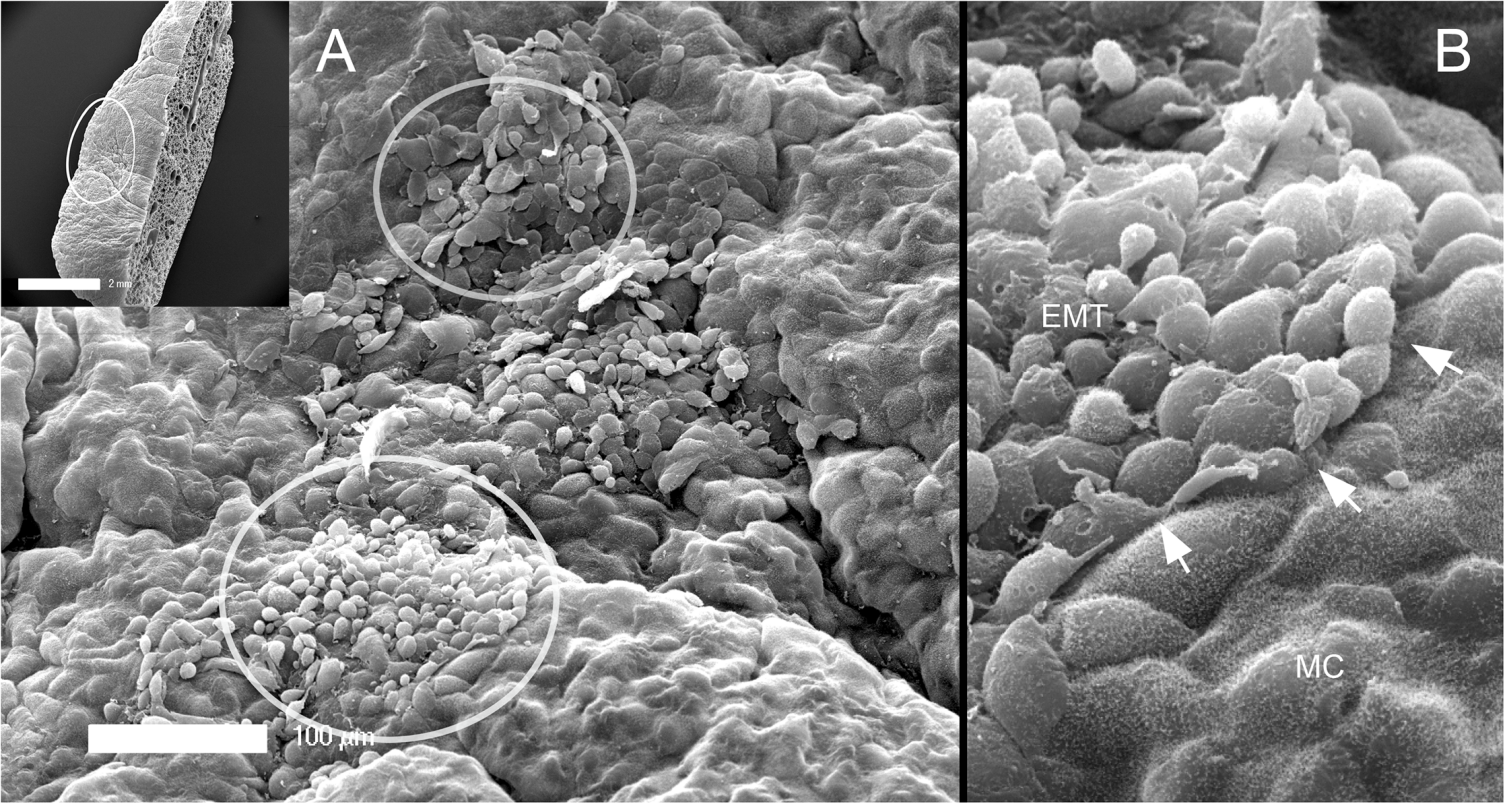
Scanning electron microscopy (SEM) of the post-operative pleural mesothelium. SEM of the cardiac lobe is shown 3 days after pneumonectomy (inset). A) Distinct morphologic forms of epithelial-to-mesenchymal transition (EMT) was seen (circles). B) There were clear demarcations between transitional zones and normal mesothelial cells (MC) with intact microvilli (arrows).

**Fig 4:**
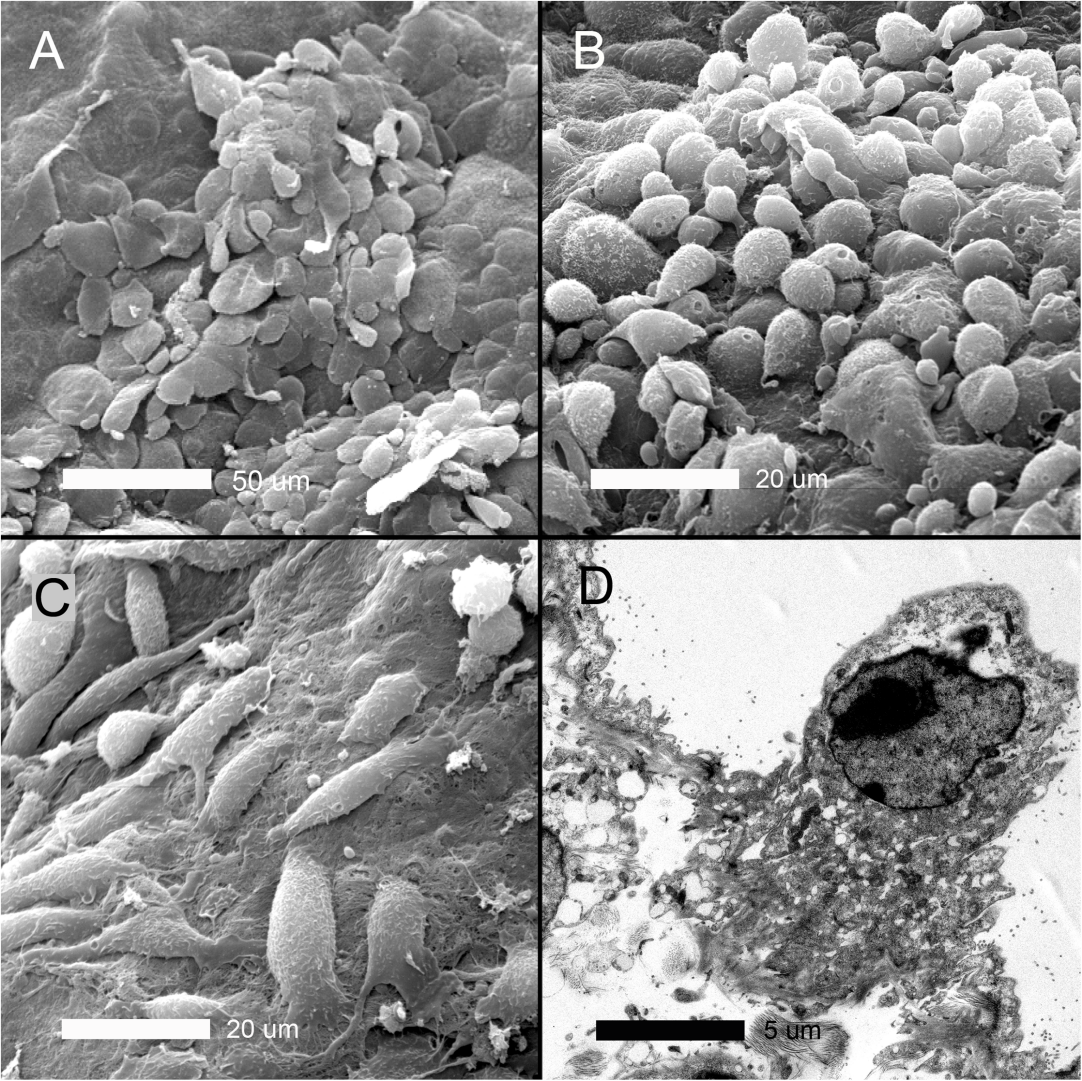
Electron microscopy of the cardiac lobe pleural mesothelium 3 days after pneumonectomy. A) Cells apparently in the early stages of transition—with disruption of intercellular junctions and loss of microvilli—retained the typical “flagstone” mesothelial morphology. B) Cells apparently in the later stages of transition demonstrating few intercellular junctions and rounded morphology. C) Spindle cell or fusiform morphotypes were found in some regions associated with exposed basement membrane. D) Transmission electron microscopy demonstrated mesothelial cells with Golgi and mitochondrial ultrastructure suggesting significantly enhanced metabolic activity. In some cells, TEM demonstrated chromatin condensation suggesting apoptosis in a subset of pleural cells (not shown).

To demonstrate the morphologic changes associated with the presumed pleural epithelial-mesenchymal transition (EMT), SEM was performed of the visceral pleura 3 days after pneumonectomy. Well-defined areas of EMT were observed (circles) with two distinctive morphologic features (Fig 3). Some areas demonstrated disruption of intercellular junctions and loss of microvilli; however, these cells retained the flat mesothelial morphotype (Fig 4A). Other cells demonstrated no intercellular junctions and rounded morphology (Fig 4B). Spindle cell morphotypes were found in some regions of exposed basement membrane (Fig 4C). Consistent with enhanced metabolic activity associated with EMT, transmission electron microscopy demonstrated mesothelial cells with prominent Golgi apparatus and numerous mitochondria.

### Pleural ingression

**Fig 5:**
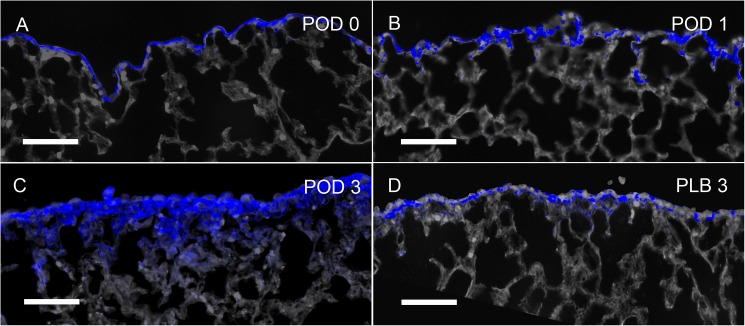
Expression of biotin in the cardiac lobe visceral pleura after pneumonectomy. The pleura was labeled with sulfo-NHS-biotin at the time of surgery. The biotin tracer was subsequently detected in tissue sections using near-infrared Quantum Dots (blue). Within 4 hours of pneumonectomy (POD 0), the biotin tracer was still largely restricted to the pleura. Hoechst 33342 nuclear stain and septal microarchitecture are pseudocolored gray. B) Within 24 hours of pneumonectomy (POD1), cells expressing biotin (blue) were detected in the pleura and contiguous alveolar septa. C) Within 3 days of pneumonectomy (POD 3), broader staining of the biotin tracer was noted extending into the subpleural septa. D) In contrast, mice 3 days after pneumonectomy and plombage (PLB) demonstrated discontinuous biotin tracer largely restricted to the pleura. Representative images shown; bar = 200 μm.

**Fig 6:**
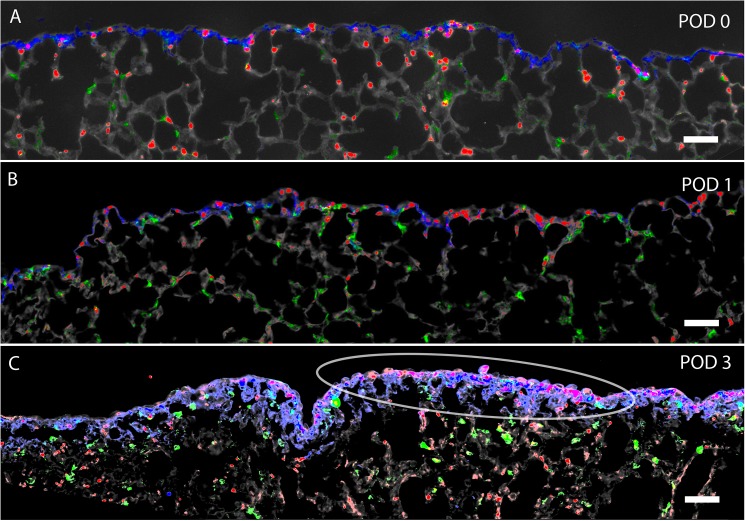
Expression of biotin, SMA, and vimentin in the cardiac lobe after pneumonectomy. The pleura was labeled with sulfo-NHS-biotin at the time of surgery. The biotin tracer was subsequently detected in tissue sections using near-infrared Quantum Dots (blue). A) Within 4 hours of pneumonectomy (POD 0), the biotin tracer was largely restricted to the pleura. Counterstaining with anti-SMA (red) and anti-vimentin (green) demonstrated scattered actin and intermediate filament staining in the pleura and subpleural alveoli. Hoechst 33342 nuclear stain and septal microarchitecture are pseudocolored gray. B) Within 24 hours of pneumonectomy (POD1), cells expressing biotin (blue), SMA (red) and vimentin (green) were detected in the pleura and contiguous alveolar septa. C) Within 3 days of pneumonectomy (POD 3), broader staining of the biotin tracer was noted. Biotin, SMA and vimentin labeling was noted deeper in subpleural alveoli. Representative images shown; pseudocolors chosen for contrast enhancement. Bar = 100 μm.

To track cell migration into the lung, the visceral pleura was labeled with a biotin tracer at the time of surgery. The lung was harvested at various times after pneumonectomy (days 0, 1, and 3) and compared to sham thoracotomy or pneumonectomy-plus-plombage controls (Fig 5). Whole-lobe image cytometry was performed to localize the biotin tracer as well as the EMT markers SMA and vimentin. Within hours of pneumonectomy, the biotin tracer was localized to the pleura with limited evidence of EMT-related migration (Fig 6A). By POD 3, biotin tracer was detected in the subpleural alveoli (Fig 6C). Image cytometry of the subpleural alveoli demonstrated a 40-fold increase in biotin^+^ cells over plombage and POD 0 controls (p < .01)(Fig 6D); 16% of all cells in the subpleural alveolar septa were biotin^+^. The migrating SMA^+^ cells did not co-express the epithelial markers E-cadherin, occludin or claudin 5. Of note, the geometry of the POD 3 alveolar ducts, in the region of migrating SMA+ cells, was indistinguishable from the post-pneumonectomy D_in_ and D_out_ dimensions previously reported [7].

**Fig 7:**
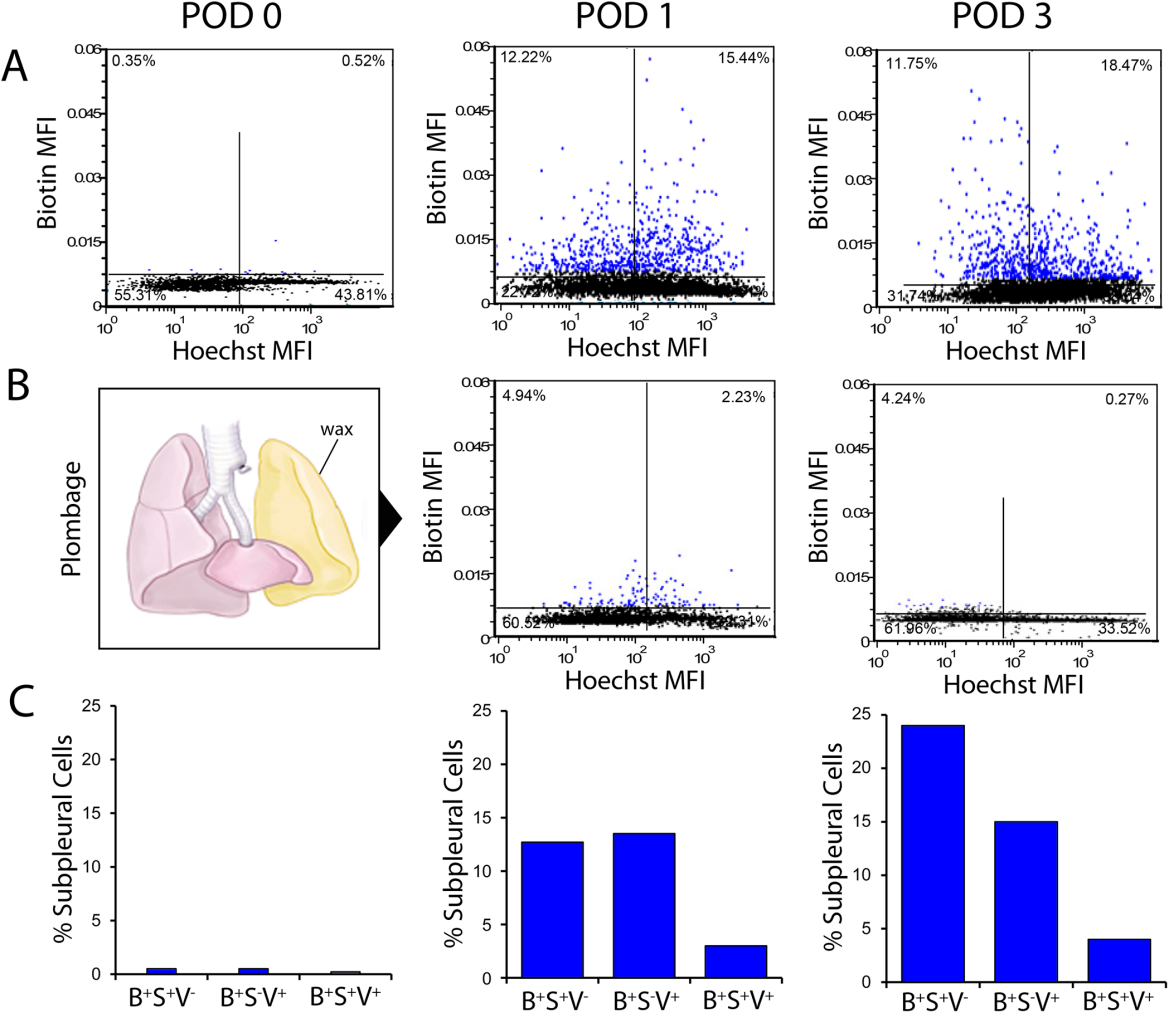
Image cytometry of the cardiac lobe after pneumonectomy. The remaining lung was labeled with sulfo-NHS-biotin intraoperatively. Columns represent whole-lobe fluorescence histochemistry and image cytometry of the cardiac lobe 4 hours (postoperative day 0, POD 0), 24 hours (POD 1) and 3 days (POD 3) after pneumonectomy. Biotin was detected with IR-avidin. Hoechst 3342 was used as a cell identifier in multiwavelength cell scoring [11] and as a semi-quantitative measure of DNA content [18]. In dual parameter dot plots, subpleural cells are colored blue. A) Dual parameter histograms reflect the relative mean fluorescence intensity (MFI) of the IR-avidin/biotin and Hoechst 3342 for individual cells in the subpleural lung detected by image cytometry. Aggregate data for the cardiac lobe in N = 3 animals is shown in each histogram. B) Plombage involved placing inert wax (volume equal to the measured lung volume change after pneumonectomy) into the empty hemithorax immediately after pneumonectomy. A comparison of subpleural cells expressing IR-avidin/biotin after pneumonectomy (A) and pneumonectomy plus plombage (B) on POD 1 and POD 3 was highly significantly (p < .01). C) Image cytometry of all subpleural cells (greater than 10^4^ cells per mouse; N = 3 mice) demonstrated that few cells expressed either SMA (3.4%) or vimentin (3.6%); however, gating on subpleural biotin^+^ cells demonstrated 29.2% of POD 1 and 38.1% of POD 3 cells expressed SMA, vimentin or SMA/vimentin. With spatial gating on subpleural lung, the cytometry results for cells expressing biotin (B^+^), SMA (S^+^) and vimentin (V^+^) are shown. Variance between mice was less than 5%.

Image cytometry of all subpleural cells in the cardiac lobe demonstrated that few cells expressed either SMA (5.4%) or vimentin (5.6%); however, gating on subpleural biotin^+^ cells demonstrated 26.2% of POD 1 and 30.3% of POD 3 cells expressed SMA, vimentin or SMA/vimentin (Fig 7). Suggesting a similar origin in space and time, the distribution of cells expressing biotin, SMA, or vimentin demonstrated a strong spatial autocorrelation in the subpleural lung (p < .001).

## Discussion

EMT involves alterations in epithelial architecture, intercellular adhesion, cellular morphology and cell migration [19]. Here, we demonstrate the expression of cytoskeletal proteins SMA and vimentin in the post-pneumonectomy visceral pleura. The changes in cytoskeletal proteins appeared to be induced by post-surgical deformation as plombage and phrenic nerve transection inhibited these changes. The increased expression of cytoskeletal proteins was coincident with the development of "patchy" areas of mesothelial transition associated with loss of microvilli, disruption of intercellular junctions, and changes in cell shape. The shape changes included the transition of the flat and polygonal mesothelial cells to cells with rounded and fusiform morphology. Finally, the transitional cells demonstrated migratory capacity as they were tracked from the pleura to subjacent alveoli. We conclude that post-pneumonectomy compensatory lung growth involves epithelial-mesenchymal transition (EMT) with the migration of transitional mesothelial cells into remodeling alveoli.

EMT is characterized by dramatic shifts in protein expression [20]. EMT has been linked to an increase in the expression of proteins such as vimentin, α-smooth muscle actin, and N-cadherin, but a decrease in abundance of E-cadherin, occludins and claudins. Because of our focus on the morphologic changes in pleural cells during EMT, we used cytoskeletal changes in actin filaments (SMA) and intermediate filaments (vimentin) to track EMT after pneumonectomy. The expression was most prominent between day 1 and 3 after pneumonectomy. Consistent with finite element model predictions of pleural deformation [21,22], EMT was both heterogeneous and concentrated in regions of the cardiac lobe characterized by a positive curvature.

A predicted feature of pleural EMT is the migration of transitional mesothelial cells into the subpleural lung. The inward migration of the transitional pleural cells—reminiscent of the inward movement (ingression) of cells during gastrulation [23,24,25]—was demonstrated by in vivo labeling of the mesothelium with biotin. Sulfo-NHS-biotin is a membrane impermeant reagent that does not diffuse through normal tight junctions [26], yet efficiently labels cell surface glycoproteins [27]. Detected by IR-avidin, the biotin tracer provided an unambiguous label for tracking the centripetal migration of transitional mesothelial cells. Many of the biotin^+^ migratory cells co-expressed either SMA or vimentin; approximately 3–4% of the biotin^+^ migratory cells expressed both transitional markers. Because multiwavelength cell scoring and image cytometry required strong fluorescent signal isolation [28], we were limited to four markers including a nuclear dye, the biotin tracer and two cytoskeletal proteins (SMA and vimentin). Although our tracking with the biotin label was restricted to the first week after pneumonectomy, we predict that many of the transitional myofibroblasts, analogous to their role in lung development [9,29], eventually localize to the tips of remodeling septa.

Consistent with the heterogeneous areas of deformation predicted by finite element modeling [21,22] and corrosion casting [3,30], the mesothelial transition areas observed in this study were confined to "patchy" areas of the mesothelium. Further, the surface area of the pleural transitions was sharply defined and roughly corresponded to the size of underlying alveolar ducts or secondary lobules. We speculate that the alveolar duct dilatation observed early after pneumonectomy [7] results in local pleural stress concentrations. Transmitted by the peripheral connective tissue system [31], supra-threshold stress concentrations are likely responsible for triggering EMT. Further, lung deformation that significantly exceeds the EMT threshold may lead to cell necrosis and/or apoptosis. The potential for mechanical “over-stretch” has clinical implications for many clinical situations—ranging from donor size-matching in lung transplantation to ventilator-induced lung injury in critical care.

EMT in the pleura has several features uniquely suited for lung regeneration and repair. Pleural EMT produces transitional cells spatially positioned to contribute to growth where lung volume expansion is the greatest. In addition, transitional cells are positioned to facilitate rapid repair in a region of the body where prolonged injury can be life threatening (e.g. pneumothorax). Further, pleural transition facilitates the centripetal migration of regenerative cells, likely via interstitial fluid flows [32], to subpleural parenchymal tissues [11].

To our knowledge, the first reference to "mesothelium" was in 1890. In describing embryologic mesoderm, Minot noted that "the mesodermic cells which bound these two [coelomic] cavities assume an epithelial arrangement, and are designated as the *mesothelium"* [33]. Interestingly, Minot also noted that “the mesothelium at various points, throws off cells which are added to the mesenchyma.” Beyond Minot's focus on its mesodermal origin, it is unclear how this "epithelial arrangement" differs from typical epithelium in other regions of the body. Similar to other epithelium, pleural mesothelium is a one cell layer thick uniform array characterized by intercellular junctions and apical-basal polarity. Moreover, our data indicates that the structural and morphologic transition of the pleural mesothelium is strikingly similar to other descriptions of EMT. To emphasize these similarities, we have referred to the pleural transition as "epithelial-mesenchymal transition." Should future work demonstrate distinct features of mesothelial transition, mesothelial-mesenchymal transition (MMT) may be a more appropriate designation.

## Abbreviations

EMT: epithelial-mesenchymal transition
IR: near-infrared
MMT: mesothelial-mesenchymal transition
SEM: scanning electron microscopy
SMA: α-smooth muscle actin
TEM: transmission electron microscopy
TLC: total lung capacity

## References

[1] Hsia CCW, Berberich MA, Driscoll B, Laubach VE, Lillehei CW, Massaro C, et al (2004) Mechanisms and limits of induced postnatal lung growth. Am J Respir Crit Care Med 170: 319–343. doi: 10.1164/rccm.200209-1062ST 15280177 10.1164/rccm.200209-1062ST

[2] Butler J, Loring SH, Patz S, Tsuda A, Yablonskiy DA, Mentzer SJ. (2012) Evidence for adult lung growth in humans. N Engl J Med 367: 244–247. doi: 10.1056/NEJMoa1203983 22808959 10.1056/NEJMoa1203983 PMC3422892

[3] Konerding MA, Gibney BC, Houdek J, Chamoto K, Ackermann M, Lee G, et al (2012) Spatial dependence of alveolar angiogenesis in post-pneumonectomy lung growth. Angiogenesis 15: 23–32. doi: 10.1007/s10456-011-9236-y 21969134 10.1007/s10456-011-9236-y PMC3268013

[4] Lin M, Chamoto K, Gibney B, Lee GS, Collings-Simpson D, Houdek J, et al (2011) Angiogenesis gene expression in murine endothelial cells during post-pneumonectomy lung growth. Resp Res 12:98. 10.1186/1465-9921-12-98 PMC3199770 21794125

[5] Fehrenbach H, Voswinickel R, Michl V, Mehling T, Fehrenbach A, Seeger W, et al (2008) Neoalveolarisation contributes to compensatory lung growth following pneumonectomy in mice. Eur Respir J 31: 515–522. doi: 10.1183/09031936.00109407 18032439 10.1183/09031936.00109407

[6] Voswinckel R, Motejl V, Fehrenbach A, Wegmann M, Mehling T, Fehrenbach H, et al (2004) Characterisation of post-pneumonectomy lung growth in adult mice. Eur Respir J 24: 524–532. doi: 10.1183/09031936.04.10004904 15459128 10.1183/09031936.04.10004904

[7] Ysasi AB, Wagner W, Bennett RD, Ackermann M, Belle JM, Valenzuela CD, et al (2015) Remodeling of alveolar septa in post-pneumonectomy lung growth. Am J Physiol 308: L1237–L1244. 10.1152/ajplung.00042.2015 PMC4587600 26078396

[8] Yamada T, Suzuki E, Gejyo F, Ushiki T (2002) Developmental changes in the structure of the rat fetal lung, with special reference to the airway smooth muscle and vasculature. Arch Histol Cytol 65: 55–69. 12002611 10.1679/aohc.65.55

[9] Dickie R, Wang Y, Butler J, Schultz H, Tsuda A (2007) Postnatal physiology of alveolar myofibroblasts: Spatiotemporal distribution and quantity of alpha-SMA contractile elements and functional implication in the developing lung. J Morphol 268: 1067–1067.

[10] Mund SI, Stampanoni M, Schittny JC (2008) Developmental alveolarization of the mouse lung. Dev Dyn 237: 2108–2116. doi: 10.1002/dvdy.21633 18651668 10.1002/dvdy.21633

[11] Bennett RA, Ysasi AB, Wagner W, Valenzuela C, Tsuda A, Pyne S, et al (2016) Deformation-induced transitional myofibroblasts contribute to compensatory lung growth. Am J Physiol 312:L79–L88. 10.1152/ajplung.00383.2016 PMC5283924 27836901

[12] Gibney B, Lee GS, Houdek J, Lin M, Chamoto K, Konerding MA, et al (2011) Dynamic determination of oxygenation and lung compliance in murine pneumonectomy. Exp Lung Res 37: 301–309. doi: 10.3109/01902148.2011.561399 21574875 10.3109/01902148.2011.561399 PMC3324589

[13] Ysasi AB, Belle JM, Gibney BC, Fedulov AV, Wagner W, Tsuda A, et al (2013) Effect of unilateral diaphragmatic paralysis on post-pneumonectomy lung growth. Am J Physiol 305: 439–445. 10.1152/ajplung.00134.2013 PMC3763038 23873841

[14] Gibney B, Houdek J, Lee GS, Ackermann M, Lin M, Simpson DC, et al (2012) Mechanostructural adaptations preceding post-pneumonectomy lung growth. Exp Lung Res 38: 396–405. doi: 10.3109/01902148.2012.715364 22905715 10.3109/01902148.2012.715364 PMC4020359

[15] Gibney BC, Park M-A, Chamoto K, Ysasi AB, Konerding MA, Tsuda A, et al (2012) Detection of murine post-pneumonectomy lung regeneration by 18-FDG PET imagin. EJNMMI Res 2: 48 doi: 10.1186/2191-219X-2-48 22999160 10.1186/2191-219X-2-48 PMC3504567

[16] Dean PN, Bagwell CB, Lindmo T, Murphy RF, Salzman GC (1990) Introduction to flow cytometry data file standard. Cytometry 11: 321–322. doi: 10.1002/cyto.990110302 2340768 10.1002/cyto.990110302

[17] Andrews PM, Porter KR (1973) Ultrastructural morphology and possible functional significance of mesothelial microvilli. Anat Rec 177: 409–426. doi: 10.1002/ar.1091770307 4127780 10.1002/ar.1091770307

[18] Santisteban MS, Montmasson MP, Giroud F, Ronot X, Brugal G (1992) Fluorescence image cytometry of nuclear-DNA content versus chromatin pattern—a comparative-study of 10 fluorochromes. J Histochem Cytochem 40: 1789–1797. doi: 10.1177/40.11.1431064 1431064 10.1177/40.11.1431064

[19] Hay ED (2005) The mesenchymal cell, its role in the embryo, and the remarkable signaling mechanisms that create it. Dev Dyn 233: 706–720. doi: 10.1002/dvdy.20345 15937929 10.1002/dvdy.20345

[20] Lamouille S, Xu J, Derynck R (2014) Molecular mechanisms of epithelial-mesenchymal transition. Nat Rev Mol Cell Biol 15: 178–196. doi: 10.1038/nrm3758 24556840 10.1038/nrm3758 PMC4240281

[21] Filipovic N, Gibney BC, Kojic M, Nikolic D, Isailovic V, Ysasi AB, et al (2013) Mapping cyclic stretch in the post-pneumonectomy murine lung. J Appl Physiol 115: 1370–1378. doi: 10.1152/japplphysiol.00635.2013 23990237 10.1152/japplphysiol.00635.2013 PMC3841828

[22] Filipovic N, Gibney BC, Nikolic D, Konerding MA, Mentzer SJ, Tsuda A. (2014) Computational analysis of lung deformation after murine pneumonectomy. Comp Methods Appl Mech Eng 17: 838–844. 10.1080/10255842.2012.719606 PMC3527685 22978574

[23] Schechtman AM (1934) Unipolar ingress in triturus torosus:A hitherto undescribed movement i the pregastrular stages of a urodele: Univ. Calif. Publ. Zool.

[24] Schechtman AM (1935) Mechanism of ingression in the egg of Triturus torosus. Proc Soc Exp Biol Med 32: 1072–1073.

[25] Fink RD, McClay DR (1985) Three cell recognition changes accompany the ingression of sea-urchin primary mesenchyme cells. Dev Biol 107: 66–74. 2578117 10.1016/0012-1606(85)90376-8

[26] Ding L, Zhang YG, Tatum R, Chen YH (2011) Detection of tight junction barrier function in vivo by biotin. In: Turksen K, editor. Claudins: Methods and Protocols. pp. 91–100. 10.1007/978-1-61779-185-7_7 PMC3228968 21717351

[27] Abdi K, Li X, Mentzer SJ (1993) Semi-dry PhastTransfer detection of biotinylated cell surface molecules. Electrophoresis 14: 73–77. doi: 10.1002/elps.1150140113 8462519 10.1002/elps.1150140113

[28] Ravnic DJ, Zhang Y-Z, Turhan A, Tsuda A, Pratt JP, Huss HT. (2007) Biological and optical properties of fluorescent nanoparticles developed for intravascular imaging. Microsc Res Tech 70: 776–781. doi: 10.1002/jemt.20463 17576122 10.1002/jemt.20463

[29] Yamada M, Kurihara H, Kinoshita K, Sakai T (2005) Temporal expression of alpha-smooth muscle actin and drebrin in septal interstitial cells during alveolar maturation. J Histochem Cytochem 53: 735–744. doi: 10.1369/jhc.4A6483.2005 15928322 10.1369/jhc.4A6483.2005

[30] Ackermann M, Houdek JP, Gibney BC, Ysasi AB, Wagner W, Belle JM, et al (2013) Sprouting and intussusceptive angiogenesis in post-pneumonectomy lung growth: mechansims of alveolar neovascularization. Angiogenesis 17: 541–551. doi: 10.1007/s10456-013-9399-9 24150281 10.1007/s10456-013-9399-9 PMC4061467

[31] Wilson TA, Bachofen H (1982) A model for mechanical structure of the alveolar duct. J Appl Physiol 52: 1064–1070. 7085408 10.1152/jappl.1982.52.4.1064

[32] Rutkowski JM, Swartz MA (2007) A driving force for change: interstitial flow as a morphoregulator. Trends Cell Biol 17: 44–50. doi: 10.1016/j.tcb.2006.11.007 17141502 10.1016/j.tcb.2006.11.007

[33] Minot C-S (1890) The mesoderm and coelom of vertebrates. Am Nat 24: 877–898.

